# Diagnostic value of circulating miR-155 for breast cancer: a meta-analysis

**DOI:** 10.3389/fonc.2024.1374674

**Published:** 2024-03-25

**Authors:** Fang Wang, Jin Wang, Hongjiang Zhang, Baobao Fu, Yanshun Zhang, Qianqian Jia, Yong Wang

**Affiliations:** Department of Oncology, Anhui University of Technology First Affiliated Hospital Huainan, Huainan, Anhui, China

**Keywords:** breast cancer, miR-155, meta-analysis, microRNA, systematic review

## Abstract

**Backgrounds:**

The value of circulating microRNA (miR)-155 for breast cancer (BC) diagnosis may differ in different studies. Therefore, we conducted this systematic review and meta-analysis to evaluate the potential application of circulating miR-155 in the diagnosis of BC.

**Methods:**

Articles published before December 2023 and in English were searched in these databases: PubMed, Web of Science, Medline, EMBASE and Google Scholar. A summary of sensitivity, specificity, positive likelihood ratios (PLR), negative likelihood ratios (NLR), and diagnostic odds ratio (DOR) were calculated from the true positive (TP), true negative (TN), false positive (FP) and false negative (FN) of each study. Additionally, the summary receive-operating characteristics (SROC) curve was constructed to summarize the TP and FP rates.

**Results:**

The pooled parameters calculated were as follows: sensitivity, 0.93 (95% CI: 0.83-0.97); specificity, 0.85 (95% CI: 0.74-0.92); PLR, 6.4 (95% CI: 3.4-11.9); NLR, 0.09 (95% CI: 0.04-0.20); and DOR, 74 (95% CI: 22-247). The analysis showed a significant heterogeneity (sensitivity, I^2^ = 95.19%, *p* < 0.001; specificity, I^2^ = 95.29%, *p* < 0.001; DOR, I^2^ = 92.9%, *p* < 0.001). The SROC curve was with an area under curve (AUC) of 0.95 (95% CI: 0.93-0.97).

**Conclusion:**

Circulating miR-155 has a potential in the diagnosis of BC.

## Introduction

Breast cancer (BC) is the most common cancer in women globally, accounting for 31% of all cancers (1). The risk of developing BC over a woman’s lifetime is approximately 1 in 8 (1). It is estimated that there will be 297,790 new cases of invasive BC and 43,170 women will die from BC in the United States in 2023 (2). Almost 20% of global BC patients occurred in China, with an estimated age-standardized incidence rate of approximately 60 cases per 100,000 women in 2040 (3, 4). The incidence of BC in China has increased rapidly in recent decades. Early detection is important for a better prognosis because there are few signs and symptoms in the early stage.

Although mammography is widely considered the gold standard for breast cancer detection, it is not without its drawbacks. It is associated with pain, anxiety, and radiation exposure (5), which can deter some individuals from undergoing regular screenings. Additionally, the effectiveness of mammography is limited in women with dense breasts (6), further reducing its reliability as a screening tool. Furthermore, mammography has been found to be particularly inaccurate in patients below the age of 40, leading to underdiagnosis (7). This is concerning because the incidence of triple-negative tumors, which have worse prognostics, is higher in this age group. Early detection is crucial for improving overall cancer survival rates, as it allows for timely intervention before the cancer has a chance to metastasize (8). To overcome the limitations of imaging techniques, the analysis of biomarkers has emerged as a promising approach for early breast cancer diagnosis. Biomarkers such as the human epidermal growth factor receptor 2 (HER2), the KI-67 protein, and estrogen receptors (ERs) are commonly used for prognosis and to guide systemic treatment decisions. In recent years, miRNAs have also gained momentum as potential biomarkers for breast cancer.

MicroRNAs (miRNAs) are a class of small endogenous RNAs that are 19-25 nucleotides in length (9). MiRNAs contribute to the post-transcription regulation of target messenger RNA (mRNA) via mRNA degradation or translation repression (10). Studies have proven that miRNAs play an important role in a variety of biological processes, including inflammation, cell-cycle regulation, cell differentiation, apoptosis, and migration (11). Besides, various studies have demonstrated that miRNA dysregulation is relevant to cancer progression, such as miR-34a in myeloma and miR-145 in solid tumors (12). And miRNA has the merits such as stable quality, easy acquisition of samples and numerous sources of samples (13). Currently, miRNA biomarkers are not utilized in clinical practice due to the significant challenge of translating them from the laboratory into validated diagnostic tests. Among the miRNAs that have been found to be deregulated in BC, microRNA-21 (miR-21) and miR-155 have been identified as the most commonly associated with BC. However, it is important to note that these miRNAs are not highly specific for diagnostic purposes (14).

MiR-155 is an important oncogenic miRNAs in human cancers including in BC (15). Abnormal expression of miR-155 has been found in multiple cancers, such as lung and cervical cancer (16, 17). The expressions of miR-155 and SOCS3 were opposite in lymphoma and pancreatic cancer cells (18, 19). MiR-155 could affect the proliferation and apoptosis of bladder cancer cells via the GSK-3β/β-catenin pathway (20). These may indicate the important role of miR-155 in cancers. Recent studies have shown that the expression level of circulating miR-155 in BC tissues was significantly higher than in normal tissues, and the level of plasma miR-155 in BC patients was also significantly higher than in healthy controls (21, 22). However, the value of circulating miR-155 for BC diagnosis may differ in different studies. Therefore, we conducted this systematic review and meta-analysis to evaluate the potential application of circulating miR-155 in the diagnosis of BC.

## Methods

### Search strategy

Articles published before December 2023 and in English were searched in these databases: PubMed, Web of Science, Medline, EMBASE and Google Scholar by two researchers (Wang F and Wang J). Search terms used were: (“miR-155” OR “microRNA-155”) AND (“breast cancer”). In addition, duplicates were removed. A total of 384 articles were screened in the study.

### Selection criteria

The study included all articles based on these inclusion criteria as follows: (i) Studies that involved human patients with BC, (ii) studies in which expression of miR-155 was measured in plasma or serum. Additionally, when true positive (TP), true negative (TN), false positive (FP), and false negative (FN) regarding the diagnostic value of circulating miR-155 for BC could not be acquired or calculated from a study, the study would be excluded. Moreover, we dropped secondary processing of literature such as reviews and meta-analysis articles. Additionally, we excluded case studies without group-level statistics. Two researchers (Wang F and Wang J) independently read the abstracts and full texts.

### Data collection

Two investigators (Wang F and Zhang H.J) read titles and abstracts of articles. We collected data as follows: Author, publication years, study location, sample type, sample size, sensitivity, specificity, cut-off value, detection method and endogenous control. In each selected article, we collected TP, TN, FP and FN directly or calculated them according to the sensitivity, specificity, positive predictive value (PPV), and negative predictive value (NPV).

### Meta-analysis for studies

All the statistical analysis was conducted using STATA 12.0 software and Meta-Disc Version 1.4. A summary of sensitivity, specificity, positive likelihood ratios (PLR), negative likelihood ratios (NLR), and diagnostic odds ratio (DOR) were calculated from the TP, FP, FN, and TN of each study. Additionally, the summary receive-operating characteristics (SROC) curve was constructed to summarize the TP and FP rates (23). Q test was used to estimate heterogeneity between studies, and computed I^2^ was used to assess the amount of variation derived from heterogeneity. With invariably high heterogeneity (Q test, *p* ≤ 0.05), random effects models were used to generate a summary effect size of these studies; Inversely, fixed effects models were performed to summarize effect size in the absence of between-study heterogeneity (Q test, *p* > 0.05).

## Results

### Search results

**Figure 1:**
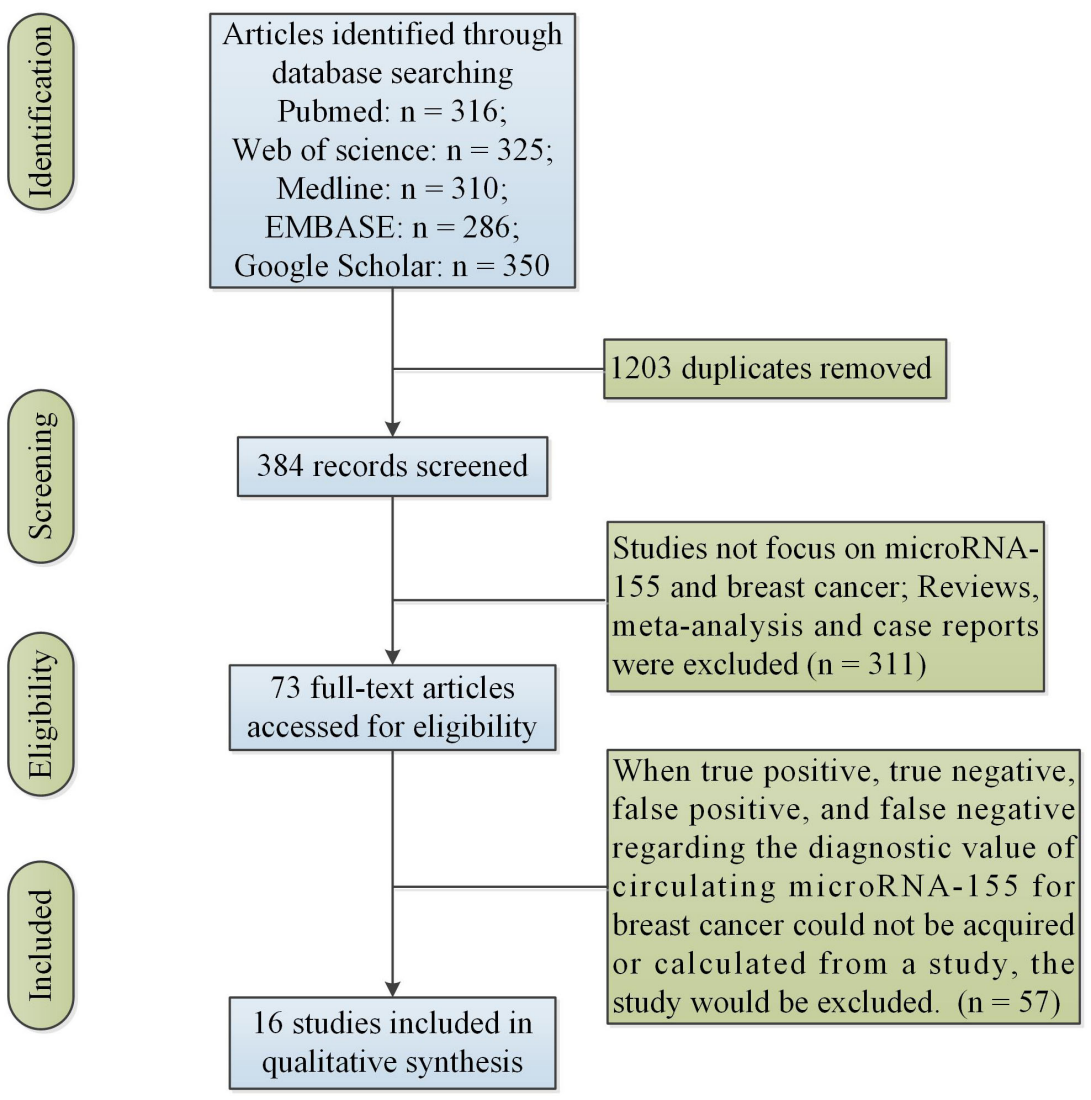
Flow of information through the different phases of a systematic review.

**Figure 1** showed the initial search results and selection process. **Table 1** showed the characteristics of the finally included 16 studies. Data were collected from 16 studies (22, 24–38) for the diagnostic studies with miR-155 for BC (BC group: n = 1,377, control group: n = 716).

**Table 1:** Characteristics of all included studies.

Reference	Country	Sample type	Sample size		Age of BC patients	Stage of BC	Sensitivity (%)	Specificity (%)	Cut-off	Detection Method	Endogenous control
			Case	Control							
Zhao et al. (2012) (24)	China	Serum	20	10	54	I: 1; II: 19	100	90	0.95	Real-time qPCR	RNU6B
Sun et al. (2012) (25)	China	Serum	103	55	51	I: 29; II: 36; III: 30; IV: 8	65.0	81.8	1.911	Real-time qPCR	cel-miR-39
Mar-Aguilar et al. (2013) (26)	Mexico	Serum	61	10	52.9	I: 13; II: 14; III: 34	94.4	100	7.92	Real-time qPCR	18S RNA
Eichelser et al. (2013) (27)	Germany	Serum	152 M0:120 M1:32	40	65	I: 69 II-IV: 51	M0: 70.6 M1: 85.3	M0: 42.7 M1:70	NR	qPCR	miR-16
Shaker et al. (2015) (28)	Egypt	Serum	100	30	25-75	NR	94.1	100	3.1585	Real-time qPCR	SNORD
Zhang et al. (2016) (29)	China	Plasma	106	106	56.9 ± 6.7	I: 11; II: 43; III: 32; IV: 20	66.0	68.9	0.321	Real-time qPCR	NR
Han et al. (2017) (30)	China	Serum	99	21	45.38	I: 49; II: 36; III: 14	100	51.02	-1.171	Real-time qPCR	NR
Fan et al. (2018) (31)	China	Serum	49	19	43	NR	100	60	NR	Real-time qPCR	miR-10b, miR-222
Huang et al. (2018) (32)	China	Serum	158	107	51.19±10.39	II-IV	Training Set: 83.3 Validation Set: 40.6	Training Set: 80 Validation Set: 87	Training Set: 1.4980 Validation Set: 0.7615	Real-time qPCR	GAPDH
Swellam et al. (2018) (33)	Egypt	Serum	80	30	52	I-II: 33III: 47		97.1	NR	Real-time qPCR	RNU6-2
Shaheen et al. (2019) (34)	Pakistan	Plasma	37	34	NR	NR	100	73.53	NR	Real-time qPCR	miR-16
Swellam et al. (2019) (35)	Egypt	Serum	96	86	50	I-II: 21III: 71	95.8	96.5	NR	Real-time qPCR	RNU6-2
Hosseini Mojahed et al. (2020) (22)	Iran	Serum	36	36	47:64 ± 8:18	I: 8; II: 17; III: 11	77.78	88.89	1.40	Real-time qPCR	SNORD47
Itani et al. (2021) (36)	Lebanon	Plasma	41	32	53 ± 11.88	0	78	75	10.54	Real-time qPCR	miR-16
Papadaki et al. (2021) (37)	Greece	Plasma	140	20	55 (27–82)	0	56.5	69.1	0.405	Real-time qPCR	NR
Mohamed et al. (2022) (38)	Egypt	Serum	99	40	48	I: 14; II: 36; III: 40; IV: 9	86.9	90	7.5	Real-time PCR	NR

GAPDH, glyceraldehyde-3-phosphate dehydrogenase; miR, microRNA; NR, not reported; qPCR, quantitative polymerase chain reaction.

### Meta-analysis results

**Figure 2:**
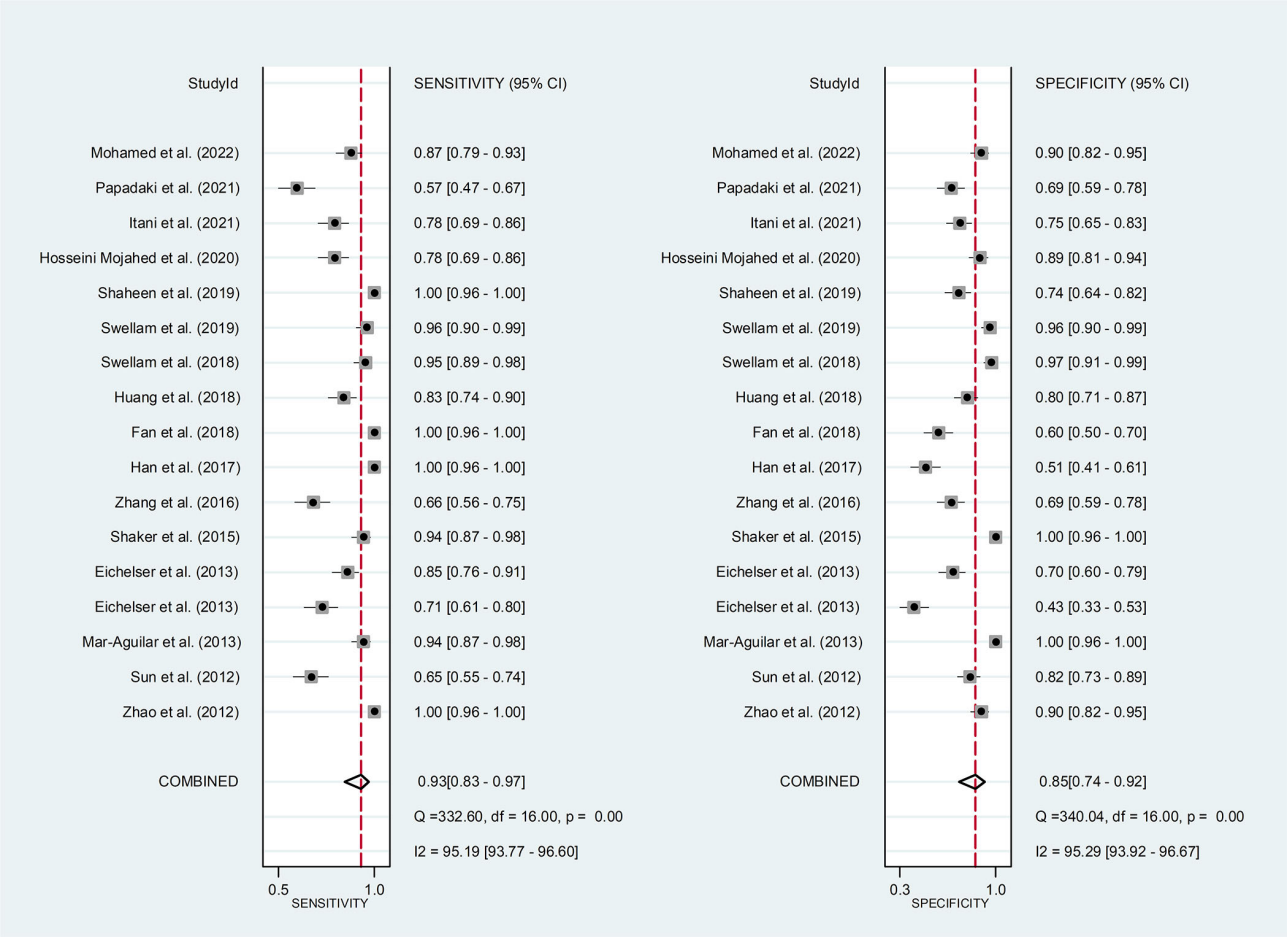
The sensitivity and specificity of circulating miR-155 in the diagnosis of BC. BC, breast cancer; miR-155, microRNA-155.

**Figure 3:**
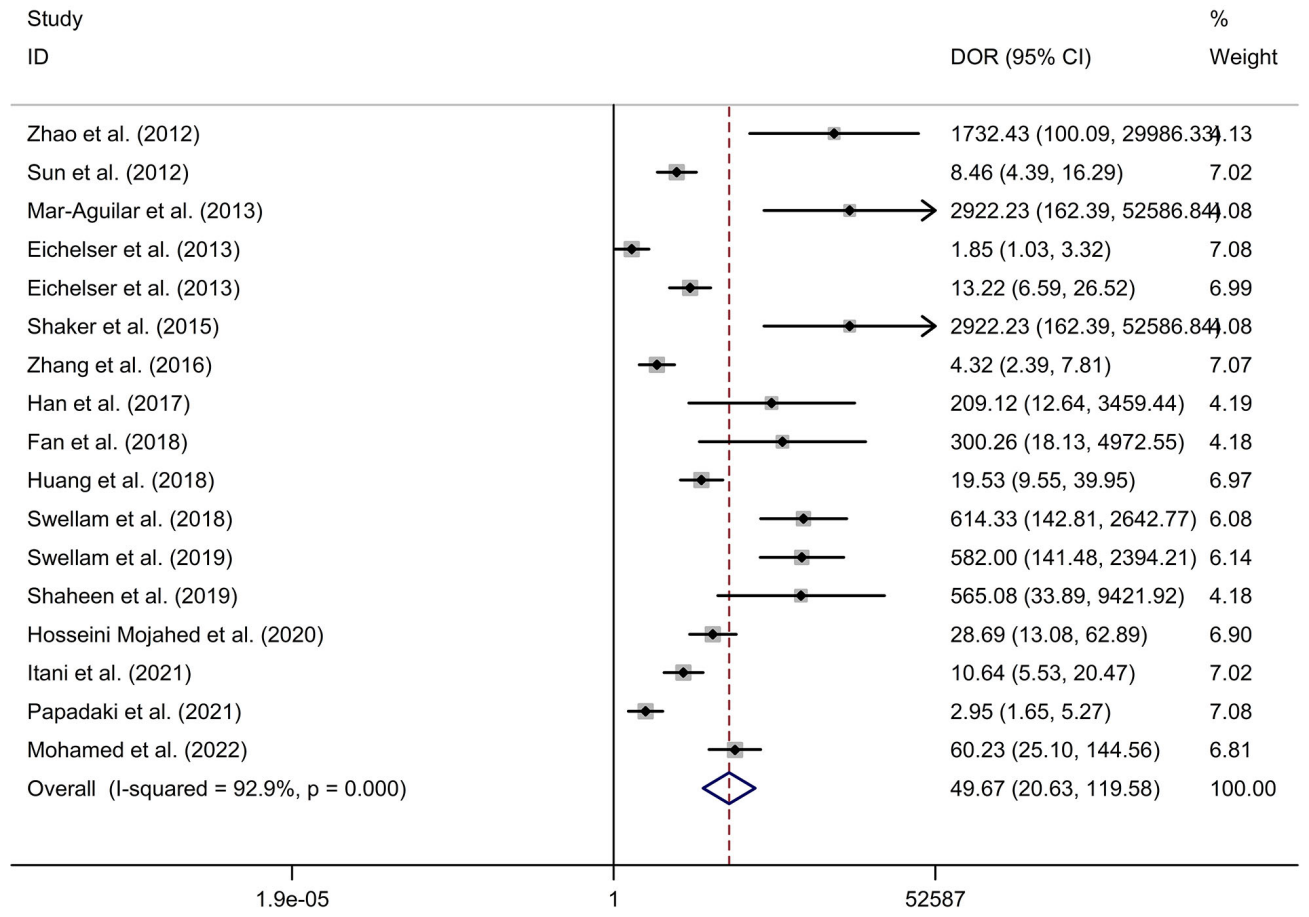
The DOR of circulating miR-155 in the diagnosis of BC. BC, breast cancer; DOR, diagnostic odds ratio; miR-155, microRNA-155.

**Figure 4:**
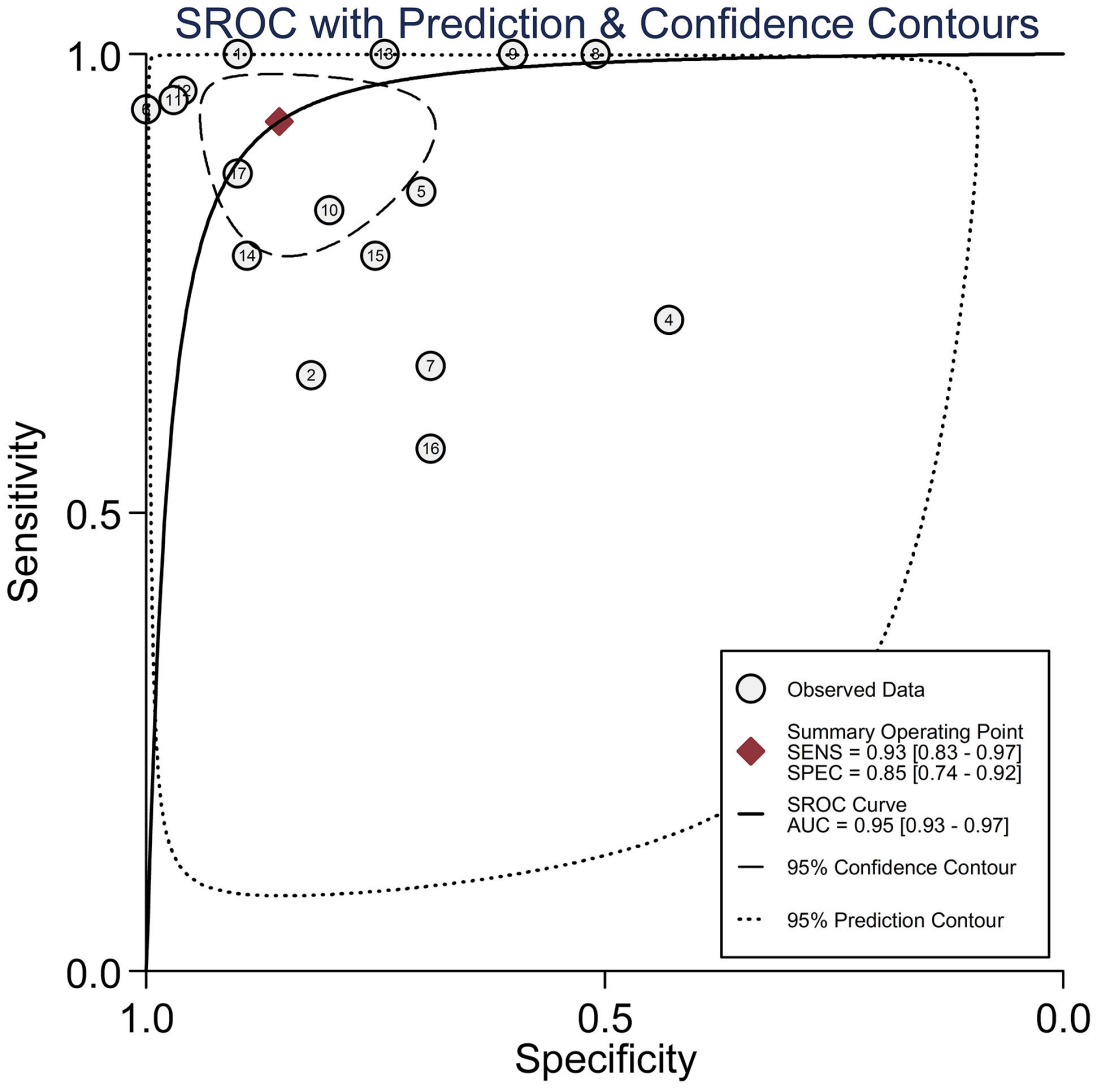
The SROC curve with AUC of circulating miR-155 in the diagnosis of BC. AUC, area under the curve; BC, breast cancer; DOR, diagnostic odds ratio; miR-155, microRNA-155; SROC, summary receiver operator characteristic.

*Circulating miR-155 showed a diagnostic value for BC.* As shown in forest plot (**Figures 2**, **3**), the pooled parameters calculated were as follows: sensitivity, 0.93 (95% CI: 0.83-0.97); specificity, 0.85 (95% CI: 0.74-0.92); PLR, 6.4 (95% CI: 3.4-11.9); NLR, 0.09 (95% CI: 0.04-0.20); and DOR, 74 (95% CI: 22-247). The analysis showed a high heterogeneity (sensitivity, I^2^ = 95.19%, *p* < 0.001; specificity, I^2^ = 95.29%, *p* < 0.001; DOR, I^2^ = 92.9%, *p* < 0.001). **Figure 4** shows the SROC curve, with an area under the curve (AUC) of 0.95 (95% CI: 0.93-0.97). The symmetrical Deek’s funnel plot showed a publication bias (*p* < 0.01, **Supplementary Figure 1**).

**Figure 5:**
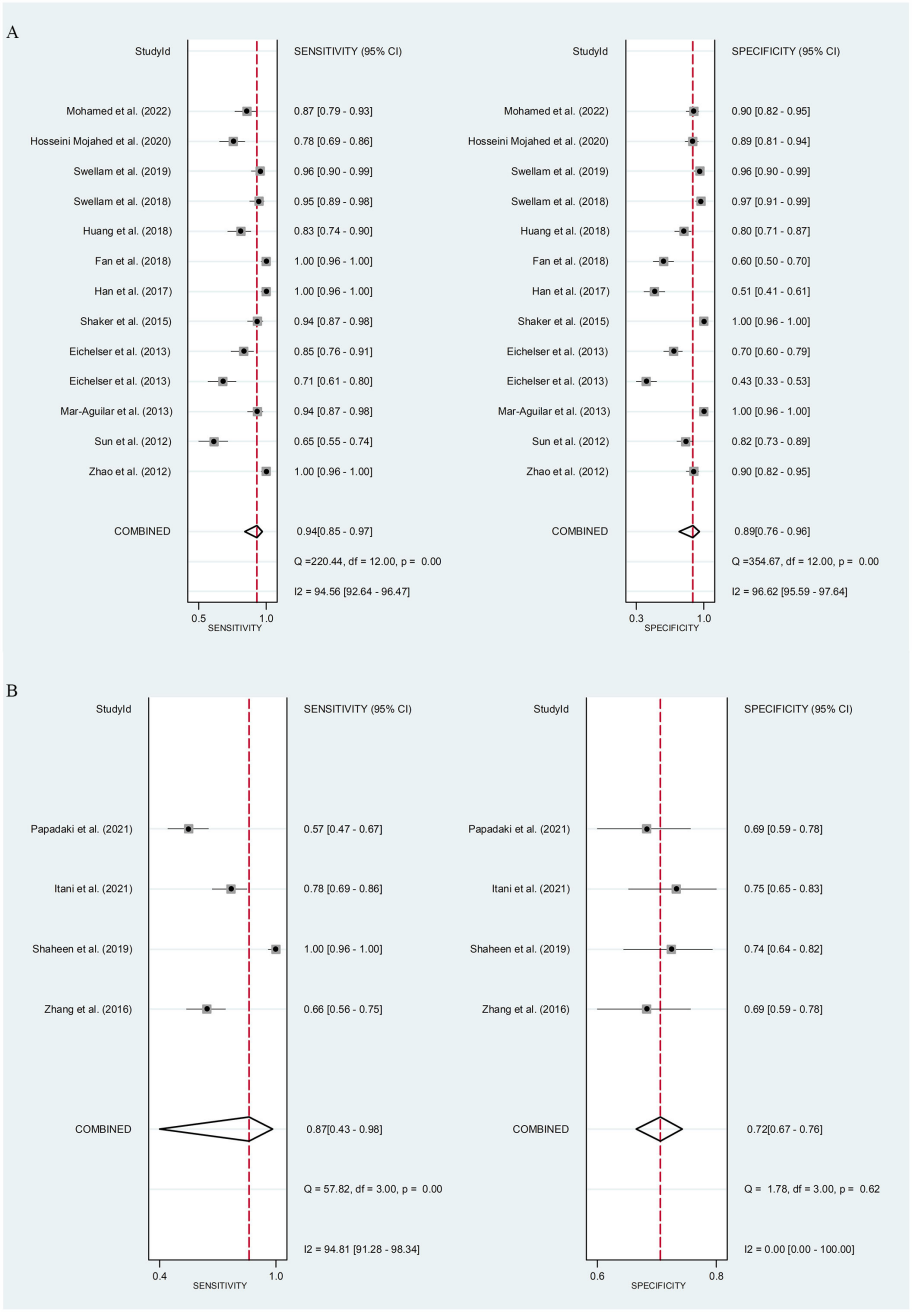
The sensitivity and specificity of serum miR-155 **(A)** or plasma miR-155 **(B)** in the diagnosis of BC. BC, breast cancer; miR-155, microRNA-155.

**Figure 6:**
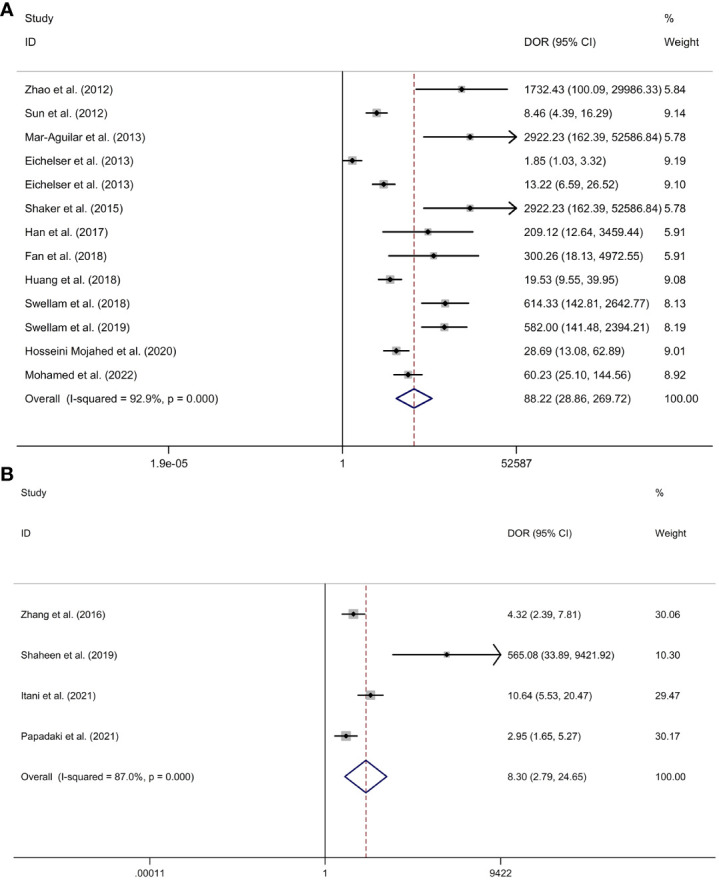
The DOR of serum miR-155 **(A)** or plasma miR-155 **(B)** in the diagnosis of BC. Abbreviations: BC, breast cancer; DOR, diagnostic odds ratio; miR-155, microRNA-155.

*Serum miR-155 showed a diagnostic value for BC.* As shown in forest plot (**Figures 5A**, **6A**), the pooled parameters calculated were as follows: sensitivity, 0.94 (95% CI: 0.85-0.97); specificity, 0.89 (95% CI: 0.76-0.96); PLR, 8.6 (95% CI: 3.6-20.4); NLR, 0.07 (95% CI: 0.03-0.17); and DOR, 123 (95% CI: 31-478). The analysis showed a high heterogeneity (sensitivity, I^2^ = 94.56%, *p* < 0.001; specificity, I^2^ = 96.62%, *p* < 0.001; DOR, I^2^ = 92.9%, *p* < 0.001). **Supplementary Figure 2** showed the SROC curve, with an AUC of 0.97 (95% CI: 0.95-0.98). The symmetrical Deek’s funnel plot showed a publication bias (*p* < 0.01, **Supplementary Figure 3**).

*Plasma miR-155 showed a diagnostic value for BC.* As shown in forest plot (**Figures 5B**, **6B**), the pooled parameters calculated were as follows: sensitivity, 0.87 (95% CI: 0.43-0.98); specificity, 0.72 (95% CI: 0.67-0.76); PLR, 3.1 (95% CI: 2.1-4.5); NLR, 0.18 (95% CI: 0.03-1.25); and DOR, 17 (95% CI: 2-163). The analysis showed a high heterogeneity for sensitivity and DOR (sensitivity, I^2^ = 94.81%, *p* < 0.001; DOR, I^2^ = 92.9%, *p* < 0.001). The analysis showed a low heterogeneity for specificity (I^2^ = 0.0%, *p* = 0.062). **Supplementary Figure 4** showed the SROC curve, with an AUC of 0.73 (95% CI: 0.95-0.98). The symmetrical Deek’s funnel plot showed a publication bias (*p* < 0.03, **Supplementary Figure 5**).

## Discussion

In our study, we found that the pooled sensitivity and specificity of circulating miR-155 were 0.93 (95% CI: 0.83-0.97) and 0.85 (95% CI: 0.74-0.92), respectively. The DOR and AUC of miR-155 were 74 (95% CI: 22-247) and 0.95 (95% CI: 0.93-0.97). A recent study showed that the sensitivity and specificity were 0.49 and 0.895 for carcinoembryonic antigen (CEA), 0.521 and 0.837 for carbohydrate antigen (CA)15-3, and the AUC of CEA and CA15-3 was 0.669 (95%CI: 0.595-0737) and 0.839 (95%CI: 0.777-0.889), respectively (35). It is widely recognized that the AUC should be in the region of 0.97 or above to demonstrate excellent accuracy (39). Compared to CEA and CA15-3, miR-155 has better sensitivity and specificity and may probably be suitable for the screening of BC. Therefore, in this meta-analysis, our result showed that miR-155 may be an excellent potential biomarker in the diagnosis of BC. The present study reported that serum miR-155 showed a high diagnostic value for BC (sensitivity, 0.94 (95% CI: 0.85-0.97); specificity, 0.89 (95% CI: 0.76-0.96); AUC, 0.97 (95% CI: 0.95-0.98)), whereas that plasma miR-155 showed a medium diagnostic value for BC (sensitivity, 0.87 (95% CI: 0.43-0.98); specificity, 0.72 (95% CI: 0.67-0.76); AUC, 0.73 (95% CI: 0.95-0.98)). The subgroup analyses based on specimen types revealed that serum had a higher diagnostic value compared to plasma, suggesting that serum may be a more suitable source of clinical specimens for BC detection. Specifically, miR-155 in serum demonstrated a more precise diagnostic value than in plasma. This discrepancy may be attributed to the coagulation process, which can influence the extracellular miRNA spectrum in the blood and consequently lead to varying miRNA expression levels in different specimens (40). Furthermore, differences in detection or normalizing methods between the two specimen types could also influence the diagnostic value (40). However, it is important to note that only four studies were included in the assessment of plasma miR-155 for BC diagnosis, which may have an impact on the overall clinical conclusion. Thus, more studies were essential to explore the diagnostic value of circulating miR-155 for BC.

The association between microRNAs and cancer has been a research focus in recent years, especially some most frequently studied microRNAs, such as miR-155. Recently published basic research results attempted to explain the association between miR-155 and the development of BC. Kim et al. reported that miR-155 was a key regulator of glucose metabolism in breast cancer via phosphoinositide-3-kinase regulatory subunit alpha (PIK3R1)-FOXO3a-cMYC axis and down-regulation of miR-155 could inhibit the growth of tumor *in vivo* (41). Wang et al. showed that miR-155 played a vital role in regulating the function of dendritic cells in BC, and miR-155 deficiency promoted BC growth in mice (42). MiR-155 was proved to play an important role in the proliferation and migration of BC cells via the down-regulation of suppressors of cytokine signaling (SOCS)1 and up-regulation of matrix metalloproteinase (MMP)16 (43). MiR-155 has also been studied as the potential prediction biomarker of early BC recurrence and therapy resistance (44, 45).

Our meta-analysis result was consistent with previous meta-analysis results published in 2014 (46). However, the previous meta-analysis only included three studies and the sample size of included studies was small. In our meta-analysis, we updated published articles regarding the association between circulating miR-155 and BC to obtain more accurate results. Some limitations still should be noticed. First, the lack of some important information from original published articles limited our research such as TNM-stage, lymph node metastasis, the level of estrogen receptor alpha (ER), progesterone receptor (PR) and human epidermal growth factor receptor (HER)-2. However, Zeng et al. reported that the expression of miR-155 was related to lymph node metastasis, and the status of ER, PR and HER-2 (47). Second, previous studies have demonstrated that circulating miR-155 was also associated with not only cancer, but also some other diseases, such as pre-eclampsia pregnancies (48), coronary artery disease (49), and multiple sclerosis (50). MiR-155-related diseases may influence the expression of miR-155 among the included samples and affect the accuracy of relevant results. So it should be noted that if we apply miR-155 as the screening BC, we should avoid the impact of miR-155-related diseases.

## Conclusions

Up until now, we can demonstrate that circulating miR-155 has a potential in the diagnosis of BC. Before circulating miR-155 can be applied to clinical diagnosis, more large-scale clinical studies should be conducted in the future.

## Data availability statement

The original contributions presented in the study are included in the article/**Supplementary Material**. Further inquiries can be directed to the corresponding author.

## Author contributions

FW: Writing – original draft, Software, Methodology, Investigation, Formal analysis, Data curation. JW: Writing – original draft, Methodology, Investigation, Data curation. HZ: Writing – review & editing, Investigation, Formal analysis, Data curation. BF: Writing – original draft, Formal analysis, Data curation. YZ: Writing – review & editing, Methodology. QJ: Writing – original draft, Methodology, Investigation. YW: Writing – review & editing, Writing – original draft, Methodology, Investigation, Formal analysis, Data curation.
