# Supplementary material for: Diagnostic value of circulating miR-155 for breast cancer: a meta-analysis
Source: Front Oncol. 2024 Mar 25;14:1374674. doi: 10.3389/fonc.2024.1374674 (PMC10999615; doi:10.3389/fonc.2024.1374674)
Supplement: Supplementary file 1 [file DataSheet_1.zip › Supplementary Figure 2.DOCX]

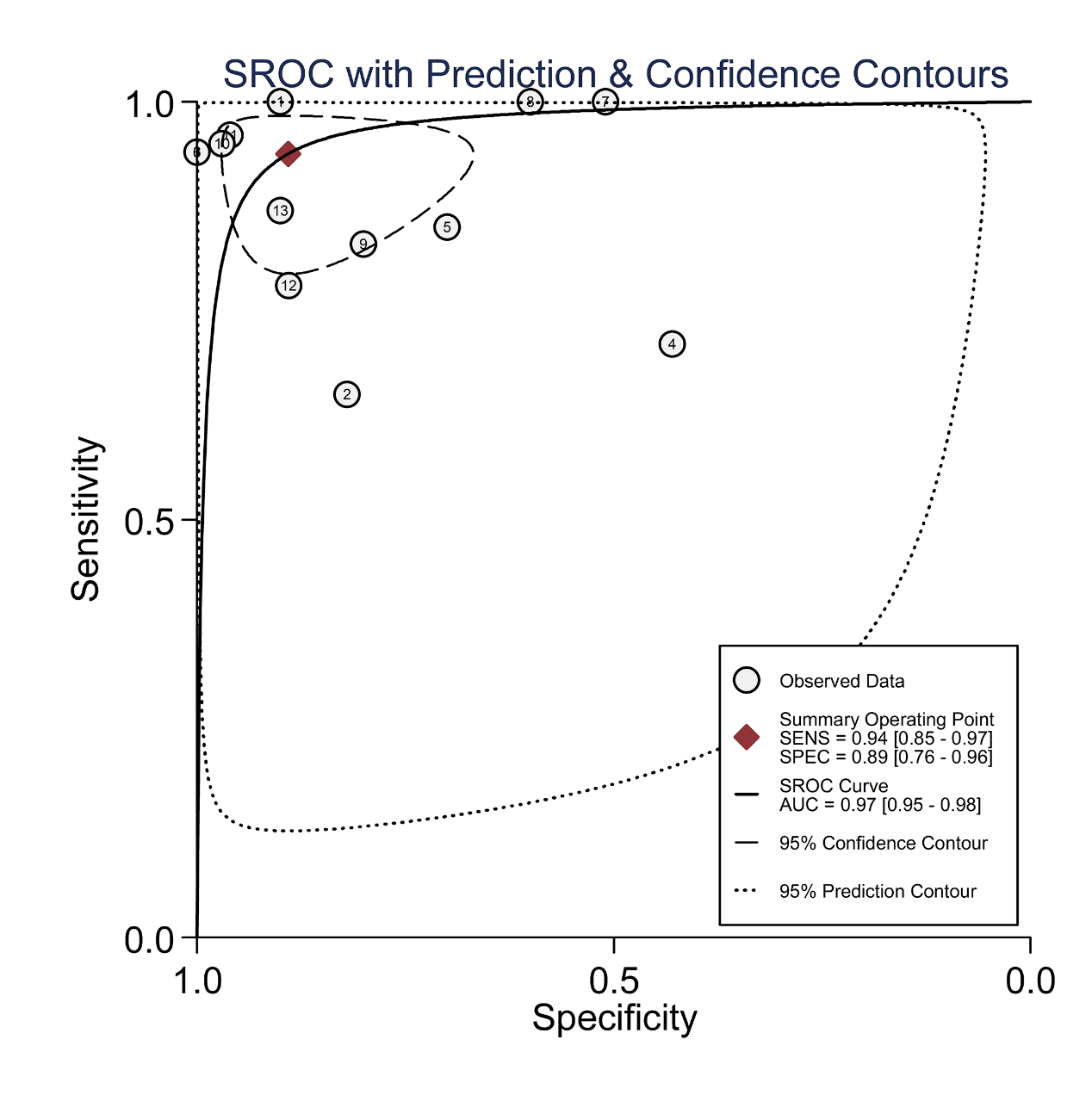


Supplementary Figure 2. The SROC curve with AUC of serum miR-155 in the diagnosis of BC. Abbreviations: AUC, area under the curve; BC, breast cancer; DOR, diagnostic odds ratio; miR-155, microRNA-155; SROC, summary receiver operator characteristic.
