# Supplementary material for: Diagnostic value of circulating miR-155 for breast cancer: a meta-analysis
Source: Front Oncol. 2024 Mar 25;14:1374674. doi: 10.3389/fonc.2024.1374674 (PMC10999615; doi:10.3389/fonc.2024.1374674)
Supplement: Supplementary file 1 [file DataSheet_1.zip › Supplementary Figure 5.DOCX]

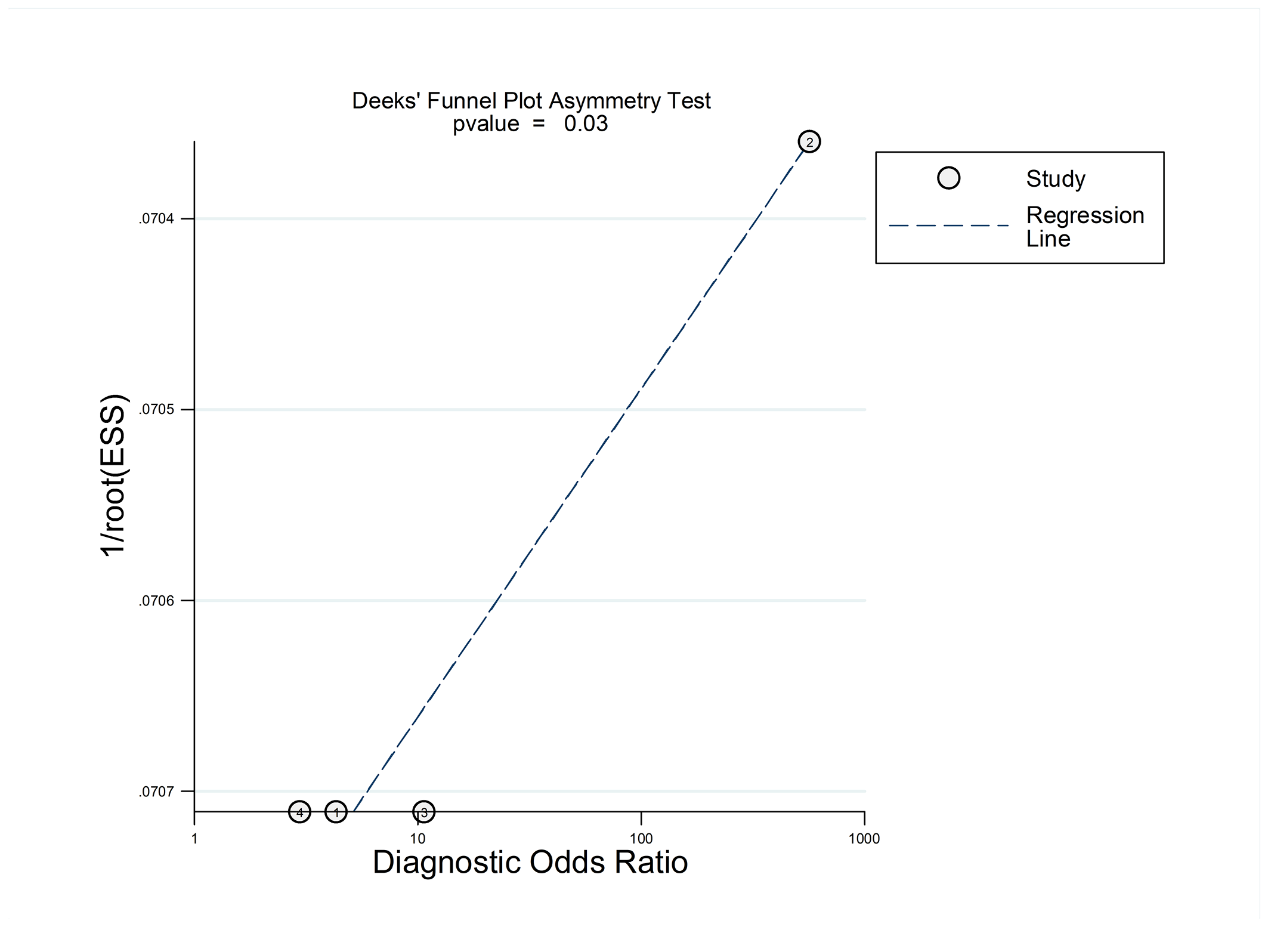


Supplementary Figure 5. Deek’s funnel plot of plasma miR-155 in the diagnosis of BC. Abbreviations: BC, breast cancer; miR-155, microRNA-155.
